# Case Report: Autologous bone marrow mononuclear cell transplantation improved motor function in a patient with amyotrophic lateral sclerosis

**DOI:** 10.3389/fmed.2026.1940826

**Published:** 2026-09-09

**Authors:** Masayoshi Okuda, Yuka Okinaka, Orie Saino, Akihiko Taguchi

**Affiliations:** 1 Department of Orthopedics, Toho Yao Hospital, Yao, Japan; 2 Department of Regenerative Medicine Research, Foundation for Biomedical Research and Innovation at Kobe, Kobe, Japan; 3 Department of Internal Medicine, Toho Yao Hospital, Yao, Japan

**Keywords:** amyotrophic lateral sclerosis, bone marrow, cell-based therapy, hematopoietic stem cell, motor function, neutrophil-to-lymphocyte ratio

## Abstract

Amyotrophic lateral sclerosis (ALS) is a neurodegenerative disease characterized by progressive motor neuron loss, and no established treatment restores impaired motor function. In mouse models of ALS, bone marrow mononuclear cell (BM-MNC) injection improved motor function. In patients with ALS, direct spinal cord injection of BM-MNCs has also been reported to improve motor function. Here, we report the case of a 52-year-old male patient with ALS who underwent intravenous autologous BM-MNC transplantation and demonstrated improved motor function. His revised ALS Functional Rating Scale score increased from 17 before treatment to 18 at 24 weeks after transplantation. The score of Manual Muscle Testing of fifth finger metacarpophalangeal joint flexion improved from 0 before treatment to 2(-) at 24 weeks after transplantation. No serious adverse events were observed. As this is a single case report, additional case series and subsequent randomized clinical trials are essential to clarify the effect of BM-MNC transplantation on ALS outcomes. This case suggests, however, that motor dysfunction in patients with ALS might be reversible. These findings encourage future studies investigating motor function improvement, rather than disease progression slowing, by elucidating the mechanisms underlying BM-MNC transplantation and optimizing treatment.

## Introduction

Amyotrophic lateral sclerosis (ALS) is an adult-onset neurodegenerative disease characterized by progressive motor neuron loss, and no established treatment restores impaired motor function (1). Although the underlying causes of ALS remain debated (2), proposed mechanisms include oxidative stress with mitochondrial dysfunction (3), astrocyte-mediated neurotoxicity (4), blood-spinal cord barrier dysfunction (5), and impaired glycolysis in the nervous system (6). Bone marrow mononuclear cells (BM-MNCs) are enriched in hematopoietic stem cells (7). Intravenously transplanted BM-MNCs directly interact with endothelial cells and activated astrocytes through gap junctions (8), activating glycolysis via hypoxia inducible factor 1*α* (Hif1*α*) (9). BM-MNC injection improved motor function in mouse models of ALS (10), and direct spinal cord injection improved motor function in patients with ALS (11). Here, we report a male patient with ALS who underwent intravenous autologous BM-MNC transplantation and showed motor function recovery.

## Case description

A 52-year-old male patient with ALS underwent intravenous autologous BM-MNC transplantation. He developed right-hand weakness in 2021 and was diagnosed with ALS in 2022. He participated in a clinical trial of bosutinib between 2022 and 2023 (12). Motor function continued to decline, and he started non-invasive positive pressure ventilation (NPPV) in 2024. He had a history of playing American football and no family history of ALS. Genetic testing of superoxide dismutase 1 (SOD1) detected no ALS-related mutations (13), and no pathological diagnosis was established. Despite treatment with Riluzole, Edaravone, and Mecobalamin, motor function continued to decline. His revised ALS Functional Rating Scale (ALSFRS-R) (14) score was 17 before treatment, and he was unable to move any fingers before transplantation.

He underwent autologous BM-MNC transplantation in October 2025. The treatment was performed according to the Act on the Safety of Regenerative Medicine in Japan (15). The protocol was approved by the Certified Special Committee for Regenerative Medicine and registered in the List of Regenerative Medicine Treatment Plans in Japan (https://saiseiiryo.mhlw.go.jp/published_plan/index/2, Protocol No. PB5250085). The procedure followed our previous protocol for autologous BM-MNC transplantation in patients with stroke (16). Briefly, 50 mL of autologous bone marrow was obtained from the posterior iliac bones using a 13-gauge bone marrow aspiration needle (Argon medical device, TX, USA) under local anesthesia. A total of 500 units of heparin (Mochida, Tokyo, Japan) was added to prevent bone marrow coagulation, followed by dilution with 150 mL of RPMI 1640 medium (Cat. No.11835030; Thermo Fisher Scientific, MA, USA). BM-MNCs were isolated by density-gradient centrifugation using Ficoll (Ficoll-Paque PREMIUM; Cytiva, MA, USA) at 400 × *g* for 40 min. Cells in buffy coated fraction were isolated and washed thrice with RPMI 1640 containing 2% of human albumin (Japan Blood Products Organization, Tokyo, Japan). A total of 5 × 10^8^ autologous BM-MNCs in 20 mL of RPMI 1,640 containing 2% human albumin was infused intravenously over 10 min using an infusion pump approximately 3 h after bone marrow harvesting. The viability rate was over 95% and the number of CD34-positive cells, a maker of hematopoietic stem cells, was quantified according to International Society for Hematotherapy and Graft Engineering (ISHAGE) guidelines using anti-CD45 and anti-CD34 antibodies (Stem Cell Enumeration kit: BD Bioscience, NJ, USA). CD34-positive cells comprised 2.5% of the transplanted BM-MNCs, corresponding to approximately 1.3 × 10^7^ cells.

Medication and rehabilitation remained unchanged after cell transplantation. The patient reported no therapeutic effect during the first month after transplantation. However, 8 weeks after transplantation, he noticed subtle recovery of right-finger motor function, which he had never experienced since disease onset. Notably, muscle fasciculations, previously observed throughout the body, disappeared 4 weeks after transplantation. Motor function further improved by 16 weeks (Supplementary Video 1) and was maintained at 24 weeks (Supplementary Videos 2, 3). Finger muscle strength was assessed by Manual Muscle Testing (MMT) (17) before and at 16 and 24 weeks after cell transplantation. Peak pulling force during maximal isometric contraction was measured at 24 weeks after treatment using a finger flexor dynamometer (Shinwa Rules, Niigata, Japan) as described previously (18). Table 1 shows the sequential change of MMT scores and peak pulling force at 24 weeks after treatment. Improved motor function of the thumb and fifth finger was observed after cell transplantation. The ALSFRS-R score increased from 17 before treatment to 18 at 16 and 24 weeks after treatment. Table 2 shows the change in complete blood count. A decrease in neutrophil-to-lymphocyte ratio (19) was observed after BM-MNC transplantation. Table 3 shows the change in blood gas analysis. No changes were made to the NPPV settings during this period. Although some improvement was observed, additional cases are needed to clarify the effects of cell transplantation on respiratory function.

**Table 1:** Manual muscle testing (MMT) scores before, 16, and 24 weeks after treatment, and peak pulling force at 24 weeks after treatment.

Muscle Action		MMT score		Peak pulling force (kg)	
		Right	Left	Right	Left
Thumb	Abduction	0→1→2	0→0→1	NT	NT
	Adduction	0→0→0	0→0→0	NT	NT
	Opposition	0→0→0	0→0→0	NT	NT
	MCP and IP Extension	0→0→0	0→0→0	NT	NT
	MCP Flexion	0→0→0	0→0→0	NT	NT
	IP Flexion	0→0→0	0→0→0	NT	NT
Second Finger	MCP Flexion	0→0→0	0→0→0	NT	NT
	MCP Extension	0→0→0	0→0→0	NT	NT
	PIP and DIP Flexion	0→0→0	0→0→0	NT	NT
	Abduction	0→0→0	0→0→0	NT	NT
	Adduction	0→0→0	0→0→0	NT	NT
Fifth Finger	MCP Flexion	0→2(-)→2(-)	0→0→0	0.25	NT
	MCP Extension	0→1→1	0→0→0	NT	NT
	PIP and DIP Flexion	0→2(-)→2(-)	0→0→0	0.16	NT
	Abduction	1→2(-)→2	0→0→0	0.26	NT
	Adduction	0→2(-)→2(-)	0→0→0	0.20	NT

Each MMT is shown as the score of [before]→[16 weeks after treatment]→[24 weeks after treatment].

MCP, metacarpophalangeal joint; IP, interphalangeal joint; PIP, proximal interphalangeal joint; DIP, distal interphalangeal joint; NT, not tested.

**Table 2:** Changes in complete blood count.

Blood count	Before	Next day	4 months	6 months
RBC (×10^4^ cell/*μ*L)	409	366	416	376
Platelet (×10^4^ cell/μL)	16.0	16.2	21.6	19.0
WBC (cells/μL)	5,200	5,340	6,970	4,900
WBC differential (%)				
Neutrophils	69.2	59.2	64.4	59.6
Lymphocytes	21.5	32.0	25.5	29.6
Monocytes	5.4	6.9	8.5	9.0
Eosinophils	3.5	1.7	1.3	1.4
Basophils	0.4	0.2	0.3	0.4
Neutrophil-to-lymphocyte ratio	3.22	1.85	2.53	2.01

**Table 3:** Changes in blood gas analysis.

Blood gas	February 2025	July 2025	December 2025
pCO2 (mmHg)	53.7	49.3	46.7
pO2 (mmHg)	72.9	88.7	119.0
sO2 (%)	91.6	93.8	97.4

October 2025 BM-MNC transplantation.

## Discussion

This report describes a patient with ALS who underwent autologous BM-MNC transplantation and experienced motor function recovery accompanied by disappearance of fasciculations. As this report describes a single case, additional case studies and subsequent randomized clinical trials are needed to clarify the effect of BM-MNC transplantation on ALS outcomes. This case suggests, however, that motor dysfunction in patients with ALS might be partially reversible. The findings are consistent with a previous study showing that direct spinal cord injection of BM-MNCs improved motor function in patients with ALS (11) and encourage development of therapies aimed at improving motor function rather than only slowing disease progression.

BM-MNCs are a heterogeneous cell population comprising hematopoietic stem cells, monocytes, T cells, and B cells (20). Clinical trials of autologous BM-MNC transplantation have been conducted for limb ischemia (21), stroke (16), bone fractures (22), and liver cirrhosis (23), with encouraging results. Proposed mechanisms include cell differentiation, paracrine signaling, and exosome secretion (24). Recent studies highlight direct cellular communication through gap junctions, leading to Hif1*α* activation, a master regulator of glycolysis, in injured endothelial cells (9). Intravenously transplanted BM-MNCs also transfer small water-soluble molecules to activated glia, neural stem cells, and osteoblasts through gap junctions, although the transferred molecules and their functions remain under investigation (8, 25). ALS is characterized by progressive motor neuron loss and marked phenotypic heterogeneity (2). Although more than 50 causative or disease-modifying genes have been identified, approximately 90% of cases are sporadic (26). Recent evidence implicates impaired glycolysis (6), endothelial dysfunction (5), and activated glia (4) in ALS pathogenesis. These findings suggest that the therapeutic mechanism of BM-MNC transplantation may involve gap junction-mediated cellular interactions. In this case, gap junction mediated cellular interactions between transplanted cells and cells in the neuronal system, such as astrocyte and spinal cord/cerebral endothelial cells, or gap junction-mediated cellular potential of transplanted cells was not evaluated. Further studies are required to reveal the link between transplanted cells and injured neuronal cells in ALS.

Neutrophil-to-lymphocyte ratio has been proposed as an indicator of systemic inflammation for neurodegenerative diseases, such as Alzheimer's disease (27) and ALS (19). Elevated neutrophil-to-lymphocyte ratio in ALS is associated with worse clinical outcomes, including faster disease progression, reduced forced vital capacity, shorter survival, and increased mortality (19). In this case, decreased neutrophil-to-lymphocyte ratio was observed after BM-MNC transplantation with motor function recovery. These findings suggest that a change in neutrophil-to-lymphocyte ratio might be a biomarker for cell-based therapy. However, because this report describes a single case, further case series are needed to validate this observation. In this case, finger muscle strength was evaluated by measuring peak pulling force during maximal isometric contraction using a finger flexor dynamometer, as previously reported (18). Standardization of the methods is required to accurately assess weak muscle strength in further studies.

This case report has some limitations. Larger pilot studies and prospective randomized trials are needed to determine the efficacy of BM-MNC transplantation in ALS. The observed motor improvement was modest and did not improve activities of daily living. In addition, identification of responders, evaluation of the relationship between therapeutic effects and gap junction-mediated properties of BM-MNCs, and elucidation of the underlying mechanisms are necessary to establish this therapy. Nevertheless, this case provides new insights into ALS treatment.

## Conclusion

This case demonstrates that motor dysfunction in patients with ALS might be partially reversible; however, larger pilot studies followed by prospective randomized trials are required to validate the efficacy of BM-MNC transplantation in ALS. These findings support further studies focusing on improving motor function in patients with ALS.

## Data Availability

The original contributions presented in the study are included in the article/Supplementary Material, further inquiries can be directed to the corresponding author.

## Supplementary material

Supplementary Video 1 Movement of the right fifth finger at 16 weeks after cell transplantation. Although the patient could not move any fingers before treatment, movement of the right fifth finger was observed 16 weeks after cell transplantation.

Supplementary Video 2 Movement of the right fifth finger at 24 weeks after cell transplantation. The patient remained able to move the right fifth finger 24 weeks after cell transplantation.

Supplementary Video 3 Movement of the right thumb at 24 weeks after cell transplantation. Movement of the right thumb was observed 24 weeks after cell transplantation.
